# Quantifying the Effects of Vegetation Restorations on the Soil Erosion Export and Nutrient Loss on the Loess Plateau

**DOI:** 10.3389/fpls.2020.573126

**Published:** 2020-11-27

**Authors:** Jun Zhao, Xiaoming Feng, Lei Deng, Yanzheng Yang, Zhong Zhao, Pengxiang Zhao, Changhui Peng, Bojie Fu

**Affiliations:** ^1^State Key Laboratory of Urban and Regional Ecology, Research Center for Eco-environmental Sciences, Chinese Academy of Sciences, Beijing, China; ^2^Institute of Soil and Water conservation, Northwest A&F University, Yangling, China; ^3^College of Forestry, Northwest A&F University, Yangling, China; ^4^Department of Biology Sciences, Institute of Environment Sciences, University of Quebec at Montreal, Montreal, QC, Canada

**Keywords:** soil erosion, nutrient loss, Loess Plateau, Grain for Green Project, vegetation restoration

## Abstract

The transport of eroded soil to rivers changes the nutrient cycles of river ecosystems and has significant impacts on the regional eco-environment and human health. The Loess Plateau, a leading vegetation restoration region in China and the world, has experienced severe soil erosion and nutrient loss, however, the extent to which vegetation restoration prevents soil erosion export (to rivers) and it caused nutrient loss is unknown. To evaluate the effects of the first stage of the Grain for Green Project (GFGP) on the Loess Plateau (started in 1999 and ended in 2013), we analyzed the vegetation change trends and quantified the effects of GFGP on soil erosion export (to rivers) and it caused nutrient loss by considering soil erosion processes. The results were as follows: (1) in the first half of study period (from 1982 to 1998), the vegetation cover changed little, but after the implementation of the first stage of the GFGP (from 1999 to 2013), the vegetation cover of 75.0% of the study area showed a significant increase; (2) The proportion of eroded areas decreased from 41.8 to 26.7% as a result of the GFGP, and the erosion intensity lessened in most regions; the implementation significantly reduce the soil nutrient loss; (3) at the county level, soil erosion export could be avoided significantly by the increasing of vegetation greenness in the study area (*R* = −0.49). These results illustrate the relationships among changes in vegetation cover, soil erosion and nutrient export, which could provide a reference for local government for making ecology-relative policies.

## Introduction

Soil erosion export is the part of eroded soil that transported to rivers. It is commonly calculated as the difference between the removal of eroded soil and the deposition of new soil (Yue et al., 2016). Soil erosion, the main process of soil erosion export, occurs naturally but is accelerated by human activities. The physical reservoir of carbon held in soil aggregates could be destroyed by soil erosion (Yue et al., 2016; Deng et al., 2018), resulting in a decrease in effective root depth, nutrient availability and water holding capacity in the root zone (Yang et al., 2003; Du et al., 2019). Soil erosion removes considerable quantities of topsoil, which will greatly influence soil nutrient stocks and will impact soil nutrient redistribution and global biochemistry (Quinton et al., 2010). However, accurately evaluating the soil erosion and its export to rivers is not an easy thing, due to the complex interaction that occur in the horizontal directions, such as temporary and longer-term sediment sinks (Piao et al., 2009; Maher et al., 2013; Taillardat et al., 2018). Besides, scale effects also have a big influence in measuring. Up to now, few retrospective assessments have been made to measure the amount of the retained soil especially the subsequently reduced soil nutrients losses by vegetation restoration for a whole period at the catchment scale. Hence, accurately assessing the processes of nutrient exchange is critical for tracking nutrient migration and for studying its effects on other systems (Bouwman et al., 2013).

Soil erosion and soil erosion export are affected by many factors, including climate, topography, vegetation cover, root systems, soil properties and land management practices (Renard et al., 1997; Fu et al., 2010). Soil erosion is usually quantified by the revised universal soil loss equation (RUSLE) (Renard et al., 1994). Compared with other factors in the RUSLE, vegetation cover is the most complex and unstable factor that influences soil erosion vulnerability, especially on complex terrain with intensive human activities (Zhou et al., 2008; Ochoa et al., 2016). Vegetation can aggregate the microstructure of soil and can also mitigate the impact of erosive-powered precipitation (Cheng et al., 2015; Guerra et al., 2016; Zhao et al., 2017a). Living plant roots modify both mechanical and hydrological characteristics of the soil matrix and contribute to soil retention (De Baets and Poesen, 2010; Vannoppen et al., 2015). The change of vegetation cover is commonly detected by NDVI, which is a comprehensive indicator to reflect the growth of plants and is directly linked to aboveground biomass. The aboveground is closely related to the belowground biomass (Oñatibia et al., 2017; Lopatin et al., 2019), which is determined the root’s ability in reducing soil erosion (Hu and Huang, 2019; Ozdemir and Yilmaz, 2020).

Nutrient cycles in terrestrial ecosystems have received increasing attention worldwide because of their emission of oxides from soils into the atmosphere that contribute to the acceleration of global warming (Jenkinson et al., 1991; Zaehle et al., 2010). Soil is the third largest global carbon stock in the terrestrial ecosystem, which ranges from 2,376 to 2,456 Pg of C in the top 2 m of soil and it is the largest contributor to N_2_O emissions (Li et al., 2014; Saikawa et al., 2014). Soil is typically the major source of plant-available phosphorus which regulates litter decomposition and soil organic carbon dynamics (Hou et al., 2018). The nutrient losses induced by soil erosion change the nutrient cycles in rivers and oceans when entering hydrological ecosystems (Bennett et al., 2001; Bauer et al., 2013) and become major environmental threats to the sustainability and productive capacity of agriculture (Yang et al., 2003). Minor changes in soil nutrients could have significant impacts on river nutrient cycles and the supply of nutrients to plants; therefore, the maintenance of nutrient stocks in the soil is essential to supporting sustainable development and regional ecological security.

As one of the best-known areas in the world, the Loess Plateau has long suffered from serious soil erosion (Zhao et al., 2013) and is a source region of sediment in the Yellow River. In 1999, the government implemented the Grain for Green Project (GFGP) on the Loess Plateau (Deng et al., 2012; Zhao et al., 2017b). As one of the most drastic land use transitions, the GFGP has provided huge ecological benefits for regulating regional climate, fixing carbon, sustaining water balance, increasing biodiversity and increasing wood prodution (Jia et al., 2014; Hua et al., 2016; Wang et al., 2017). The GFGP has also improved human living environments and promoted residents’ incomes (Feng et al., 2019). The most notable contribution of the GFGP is that significantly reduced the sediment exported to rivers (Zhou et al., 2009; Wang et al., 2018). The first stage of the GFGP was completed on the Loess Plateau at 2013 (National Forestry and Grassland Adiministration, 2017); however, the extent to which vegetation restoration (caused by the GFGP) prevents soil erosion and nutrient export is still unknown. Therefore, quantifying the direct relationships among vegetation change, soil erosion and nutrient export is urgent and indispensable for regional ecology management.

To evaluate the effects of the first stage of the GFGP (1999–2013) on reducing soil erosion and nutrient export, here, we first extracted the changes in vegetation cover before and after 1999, and then we analyzed the spatial distribution of soil erosion and nutrient loss during two periods. Finally, we quantified the relationships among soil erosion, nutrient loss and vegetation changes. The objectives of this study are to (1) detect the trends of vegetation change before and after the first stage of the GFGP; (2) analyze the spatial distributions of soil erosion export before and after the first stage of the GFGP; and (3) clarify the relationships among vegetation changes, soil erosion and nutrient loss.

## Materials and Methods

### Study Region

**FIGURE 1:**
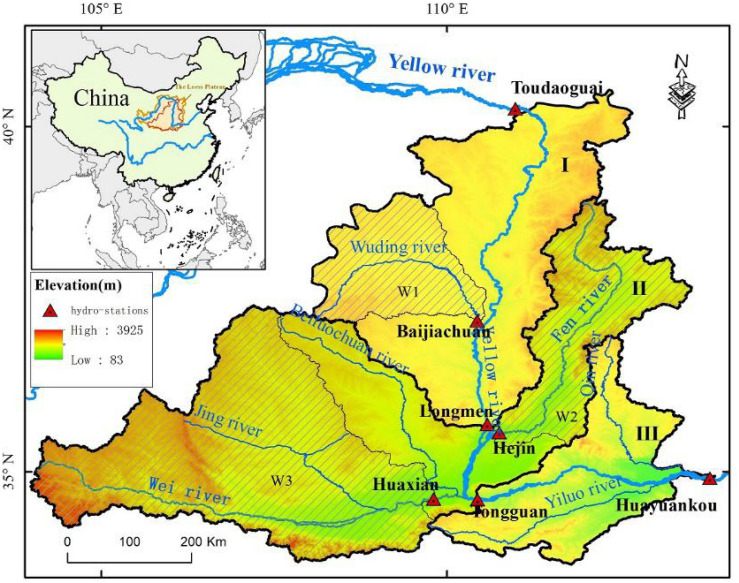
Study region. The study region was divided into three sub-catchments, including sub-catchment I: from Toudaoguai to Longmen; sub-catchment II: from Longmen to Tongguan and sub-catchment III: from Tongguan to Huangyuankou. The top-left panel shows the location of study area on the Loess Plateau. Three shaded regions are watersheds of Wuding river (W1), Fen River (W2), and Wei River (W3), respectively.

The study area is a catchment in the middle reaches of the Yellow River between the Toudaoguai and Huayuankou hydrologic stations on the Loess Plateau, with an area of 344 794 km^2^, which is considered one of most eroded areas in the world and is the main source of sediments of the Yellow River and accounts for about 54% of whole Loess Plateau (Figure 1). The study area is divided into three sub-catchments from north to south, according to the implementation intensity of the GFGP. The northern part of the study area (sub-catchment I) is the catchment between the Toudaoguai and Longmen hydrologic stations, characterized by sparse vegetation, soft loess, and high intensity rainfall in the summer, and this is the main implementation area of the GFGP. The middle part (sub-catchment II) is the catchment between Longmen and Tongguan, characterized by high-intensity human activities, i.e., crop planting and residence development, covered by the Wei River and Fen River, which are the two largest tributaries of the Yellow River. The southern part (sub-catchment III) is the catchment between the Tongguan and Huayuankou hydrologic stations characterized by intense human activities and suspended rivers.

### Data Source and Study Period

NDVI is a good indicator of surface vegetation dynamics and is directly related to the photosynthetically active radiation absorbed by canopies (Badgley et al., 2017; Duan et al., 2019). NDVI is widely acknowledged as a good indicator to represent vegetation cover and fraction (Gutman and Ignatov, 1998). Third-generation GIMMS data (GIMMS 3g, 1982–2013, 780 periods), which are widely used NDVI datasets, with a spatial resolution of 8 km and a temporal resolution of 15 days, were employed to extract the vegetation changes. Data on lateral soil erosion were calculated based on national surveys, which were carried out from 1995 to 1996 and 2010 to 2012 based on 8,980 soil profiles (Yue et al., 2016), representing the soil status before and after the GFGP. The runoff and sediment data of seven rivers used in this paper were collected from the River Sediment Bulletin of the Yellow River from 1982 to 2012. The differences in land use during the two periods (1980–2000 and 2000–2015) represent the changes before and after the GFGP, respectively. All dataset descriptions, including afforestation area per year, climate data, and soil nutrient content distributions, are summarized in Table 1.

**TABLE 1:** Dataset descriptions.

Datasets	Descriptions	Sources
GIMMS NDVI-3g	The spatial resolution is 8 km, and the temporal resolution is 15 days.	https://ecocast.arc.nasa.gov/data/pub/gimms/3g.v1/
Soil erosion data	The soil erosion data come from the Chinese national surveys from 1995 to 1996 and 2010 to 2012 at the county level.	Yue et al., 2016
Runoff and sediment data	The runoff and sediment data come from seven hydrological stations during 1982–2012.	River Sediment Bulletin of the Yellow River from 1982 to 2012
Land use type maps	The land use data are the classification results using Landsat images with a high accuracy. We use the land use data in 1980 (1 km resolution), 2000 and 2015 (90 m resolution).	Resource and environment data cloud platform, Chinese Academy of Sciences http://www.resdc.cn/
Soil nutrient content	The 0–30 cm soil nutrient content (C, N and P) in the HWSD database was used.	http://www.fao.org/land-water/databases-and-software/hwsd/en/
Afforestation area	Annual afforestation data were recorded for each county during 2002–2013.	Chinese forestry annual statistical reports
Climate data	Climate data come from 384 climatic stations on the Loess Plateau and are interpolated into 8 km resolution using the ANUSPLIN tool	National Meteorological Information Center http://data.cma.cn/

In this study, there are three research themes that all focus on the comparisons of vegetation and soil erosion export before and after the GFGP (Supplementary Figure S1). To evaluate the effects of the first stage of the GFGP (1999–2013) on reducing soil erosion and nutrient export, we separate the study period into from 1982 to 1998 and from 1999 to 2013. The analysis steps are described as sections “Detecting Changes in Vegetation and Their Drivers” to “Analysing Effects of Vegetation Restorations on Soil Erosion and Nutrient Loss.”

### Detecting Changes in Vegetation and Their Drivers

We analyzed the change trends of vegetation before and after the GFGP, and then detected their divers. The change trends of vegetation were extracted by the simple linear regression, with annual maximum NDVI as the dependent variable and the year number as independent variable. The intercepts present the trends of vegetation changes and the *P*-values determine the significant of the linear regression. Three pathways were utilized to detect the drivers of vegetation changes, especially after the GFGP. First, we compared the land use in 1980, 2000, and 2015, which presented different change features between the two periods. These results could verify the spatial consistency between the regions of the GFGP and the increased vegetation areas. Second, we analyzed the statistical data of annual afforestation areas to verify whether the vegetation in these areas had been improved. Third, we used the method of variance partition as proposed by Zheng et al. (2019) to separate the contributions of climate and human activities (mainly the GFGP) to vegetation changes. If the climate contribution exceeded 50% (Huang et al., 2016), then the area was regarded as a climate-dominated area, while areas with climatic contribution less than 25% were simply considered human activity-dominated areas; the remaining regions were dominated by the interactions of climate and human activities.

### Quantifying the Soil Erosion Export and Nutrient Loss

The soil erosion export is the part of soil erosion exported into rivers. Soil erosion export (to rivers) (F) is calculated as the difference between the removal of eroded soil (F1) and the deposition of eroded soil (F2) (Yue et al., 2016). During the two periods of 1995–1996 (standing for before the GFGP) and 2010–2012 (standing for after the GFGP), we used the Chinese national surveys of soil erosion data to calculate F1 and F2 separately at the county level. In this dataset, soil erosion grades (slight, light, moderate, intense, extremely intense and severe erosion; Table 2) and eroded area for each county on the Loess Plateau were recorded. The concrete equations of F, F1 and F2 (kg/a) at a county level are listed as follows:

(1) F=F1-F2

(2) F1=V*eroAero

(3) F=2F*1(1-SDR)

**TABLE 2:** Classification standard of soil erosion and the corresponding SDR on the Loess Plateau (Ministry of Water Resources of the People’s Republic of China, 1997).

Erosion grade	Grade description	Erosion modulus (tkm^−2^a^−1^)	Erosion rate (mma^–1^)	SDR grade
1	Slight	<1,000	<0.74	–
2	Light	1,000–2,500	0.74–1.9	0.1–0.3
3	Moderate	2,500–5,000	1.9–3.7	0.3–0.5
4	Intense	5,000–8,000	3.7–5.9	0.5–0.7
5	Extremely intense	8,000–15,000	5.9–11.1	0.7–0.9
6	Severe	>15,000	>11.1	0.9–1.0

where *V*_*ero*_ (m/a) is the water erosion rate and is termed the erosion modulus (taking the median value of the range in Table 2). *A*_*ero*_ (m^2^) is the erosion area which comes from Chinese national surveys of soil erosion for each county (Yue et al., 2016).

The sediment delivery ratio (*SDR*) is defined as the ratio of sediment transport to the total amount of soil erosion. We used five grades of soil erosion severity ranging from 0.1 to 1 generated by Jing et al. (2005) and took the median values for SDR calculations (Table 2). A low SDR indicates that more sediment (as deposition) will accumulate in the watershed.

We evaluated the soil nutrient loss before and after the GFGP at the county level by using a general model as follows:

(4) $$N_{loss}=E_{content}*F$$

where *E*_*content*_ is the nutrient content in the soil unit, which was extracted from the Harmonized World Soil Database (HWSD) (Nachtergaele et al., 2009) and averaged by 0–30 cm at the county level. F is the averaged soil erosion export at the county level as in Eq. 4.

### Analysing Effects of Vegetation Restorations on Soil Erosion and Nutrient Loss

At the county level, we quantified the changes in vegetation and soil erosion export before and after the GFGP. Before the GFGP (1990s), the soil erosion export and nutrient loss were calculated by the national survey of soil erosion at the county level during 1995–1996 (Yue et al., 2016); the vegetation changes were calculated by remote sensing data during 1982–1998 with the methods described in section “Detecting Changes in Vegetation and Their Drivers.” After the GFGP (2010s), the soil erosion export and nutrient loss were calculated by the national survey of soil erosion during the period from 2010 to 2012 (Yue et al., 2016). Vegetation changes were calculated by remote sensing data during the period from 1999 to 2013 with the same methods mentioned above. The relationship between the changes in vegetation trends and soil erosion export changes during these two periods at the county level was built by a simple linear regression.

At the catchment level, we used the changes in sediment in the rivers instead of changes in the soil erosion export. We quantified the soil erosion export and vegetation change relationship from two perspectives. First, we divided the whole study area into three sub-catchments (see section “Study Region”) and quantified the relationship before (1982–1998) and after (1999–2013) the GFGP at the catchment level. The vegetation changes (mean values of catchments) were observed by NDVI time-series data. The difference values of sediment between upstream and downstream hydrological stations were employed to represent soil erosion export. Second, we selected three typical small catchments (including the catchments of the Wuding River, Fen River and Wei River, which are controlled by the Baijiachuan, Hejin and Huaxian hydrological stations, respectively) to carry out the work described above.

## Results

### Vegetation Cover Changes

**FIGURE 2:**
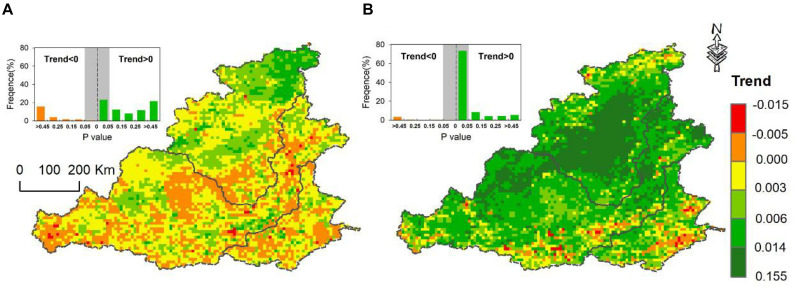
Spatial distribution of the NDVI trend before and after the GFGP. **(A)** From 1982 to 1998 (representing the 1990s). **(B)** From 1999 to 2012 (representing the 2010s).

From 1982 to 2013, the area with vegetation cover significantly increased accounted for 63.0% of the study area. Taking the implementation year of GFGP as breakpoint, obvious different trends of vegetation variations were found (Figure 2). From 1982 to 1998, before the GFGP, the vegetation changed little over time, and most of the pixels had no significant trend of vegetation changes, showing random characteristics to some extent (Figure 2A). The vegetation of sub-catchment I showed a slight improvement before the GFGP but the results were not significant in most regions. The vegetation of sub-catchments II and III did not change in most regions. However, after the implementation of the GFGP during the 1999–2012 period, vegetation showed significantly increasing trends in 73.5% of the study area (Figure 2B). The most noticeable vegetation changes occurred in catchment I, which is the main region of the GFGP implemented. The vegetation of catchment III also improved slightly compared with catchments II and I. The area of vegetation degradation accounted for approximately 30% and was mainly affected by human activities. The vegetation of northern catchment II improved, but some areas showed a trend of vegetation degradation, especially around residential areas on the Guanzhong Plain due to human activity.

### Changes of Soil Erosion Export

**FIGURE 3:**
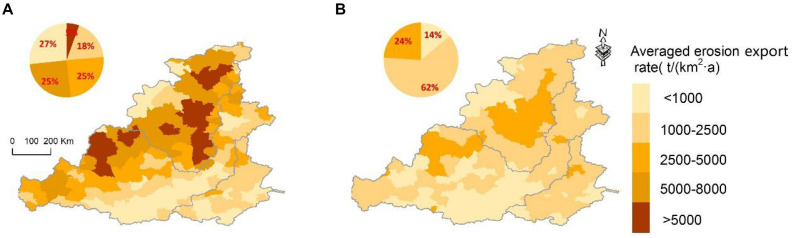
Spatial distribution of the average soil erosion export at the county level before and after the GFGP. **(A)** From 1995 to 1996 (representing the 1990s). **(B)** From 2010 to 2012 (representing the 2010s).

During 1982–2013, the patterns of soil erosion changed considerably (Figure 3). Before the implementation of the GFGP, that is in the 1990s, the eroded area was 215,981.26 km^2^, accounting for 41.8% of the study area. The counties with average erosion rates greater than 5,000 t/(km^2^a) occurred mainly in catchment I, which accounted for 24% of the study area. The average erosion rates of catchment III and southern catchment II were less than 600 t/(km^2^a). However, after the implementation of the GFGP (in the 2010s), the erosion area decreased to 137,955.33 km^2^, only accounting for 26.7% of the study area. Soil erosion export decreased, and the average rates of erosion export of all counties were less than 5,000 t/(km^2^a) (Figure 3A). The area with slight erosion [less than 1,000 t/(km^2^a)] increased from 32% to 50% of the study area (Figure 3). A significant decrease in soil erosion export occurred in catchment I. The average erosion rate of the northern part of catchment II obviously decreased, but the southern part had little change. It is worth noting that intense soil erosion export still occurred in catchment III, where the GFGP was not implemented, and human activities increased (Figure 3A).

### Changes of Soil Nutrition Loss

**FIGURE 4:**
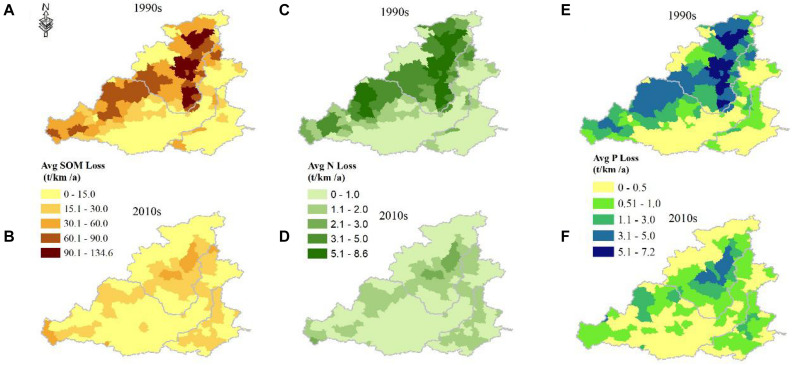
Spatial distributions of average nutrient losses in the 1990s and 2010s. **(A,B)** Average SOM loss. **(C,D)** Average soil nitrogen loss. **(E,F)** Average phosphorus loss.

Because the consistence was presented between soil erosion export and nutrient loss, the soil nutrient loss also presented a decreased trend (Figure 4). In the 1990s (before the GFGP), the regions with severe nutrient loss were distributed in catchment I and northern catchment II, where the average SOM loss exceeded 90 t/(km^2^a), the average soil N loss exceeded 5 t/(km^2^a) and the average soil P loss exceeded 5 t/(km^2^a) (Figure 4). In the 2010s, soil erosion export obviously decreased. The average SOM loss of most regions decreased to below 30 t/(km^2^a), and regions with slight erosion [less than 15 t/(km^2^a)] accounted for 64.5%. The average soil N loss of most regions decreased to below 2 t/(km^2^a), and regions with slight erosion [less than 1 t/(km^2^a)] accounted for 66.3%. The average soil P loss of most regions decreased to below 3 t/(km^2^a), and regions with slight erosion [less than 0.05 t/(km^2^a)] accounted for 84.8%.

Total nutrient losses were quantified based on the total soil erosion export and soil nutrient content. Before the implementation of the GFGP (in the 1990s), the total SOM loss was 11,596 10^3^ t/a; however, it decreased to 4,495 10^3^ t/a (a reduction of 61.2%) (Supplementary Figure S2A), and it was mainly distributed in catchment I and northern catchment II (Supplementary Figures S3A,B). The proportion of the SOM loss in catchment I decreased from 53.0% in the 1990s to 33.5% in the 2010s (Supplementary Figure S2A), indicating the strong effect of vegetation restoration in preventing soil erosion export. Total soil N losses decreased from 733 10^3^t/a in the 1990s to 274 10^3^ t/a (Supplementary Figure S2B), and catchment I was the main contributor whose proportions decreased from 53.2 to 34.9% (Supplementary Figures S3C,D). Total soil P loss also significantly decreased from 620 10^3^ t/a in the 1990s to 216 10^3^ t/a, and both catchment I and catchment II contributed to these losses (Supplementary Figures S2C, S3E,F).

### Effects of Vegetation Restorations on Soil Erosion and Nutrient Loss

**FIGURE 5:**
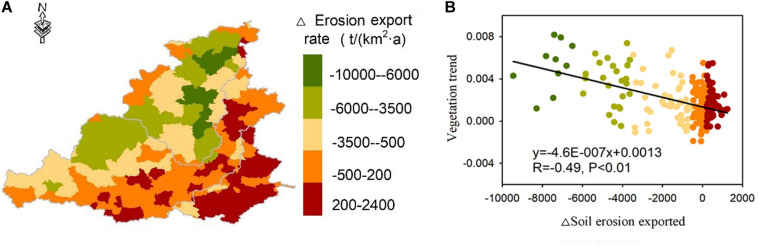
The difference in the erosion rate between the 1990s and 2010s and its relationship with the vegetation change trend. **(A)** Spatial distributions of the changes of soil erosion export rate between 2010s and 1990s. **(B)** The relationship between vegetation trend and soil erosion export changes.

Vegetation restoration effectively reduced soil erosion export (nutrient loss showed similar pattern). Changes in soil erosion export showed great spatial heterogeneity, providing an opportunity to quantify the effect of vegetation restorations on soil erosion export changes (Figure 5). The soil erosion export rates of catchment I and northern catchment II decreased significantly after the implementation of the GFGP, while the erosion export rate of the region with intense human activities increased (Figure 5A). At a county level, soil erosion export can be avoided significantly by the increasing of vegetation greenness in the study area (*R* = −0.49) (Figure 5B). The soil erosion export of areas with high vegetation trend values decreased the most (dark green points in Figure 5B), while negative vegetation trends represent severe soil erosion export (dark red points in Figure 5B).

From the perspective of spatial statistics, vegetation restorations reduced the soil erosion and nutrient loss (Table 3). In the region where erosion significantly decreased [−10000 to −6000 t/(km^2^a)], the mean erosion decrease was 7,533 t/(km^2^a), while the vegetation change was (0.53 ± 0.07) × 0.01 NDVI/a, which decreased the soil SOM loss by −85.35 ± 0.15 t/(km^2^a), decreased the soil N loss by −5.53 ± 0.01 t/(km^2^a) and decreased the soil P loss by −5.08 ± 0.01 t/(km^2^a). However, in the region in which erosion is obvious increases [200–2,400 t/(km^2^a)] and vegetation changes were not significant (*P* > 0.05 in most regions). In addition, the soil SOM loss increased by 8.83 ± 0.02 t/(km^2^a), the soil N loss increased by 0.51 ± 0.01 t/(km^2^a) and the soil P loss increased by 0.35 ± 0.001 t/(km^2^a). Soil erosion export and nutrient loss showed a covariation pattern but a positive relationship with vegetation trends.

**TABLE 3:** Relationships among the erosion rate, vegetation changes and nutrient losses at the county level.

ΔErosion rate ranges t/(km^2^⋅a)	Vegetation trend (0.01 SPOT NDVI/a)	SOM loss t/(km^2^⋅a)	Soil N loss t/(km^2^⋅a)	Soil P loss t/(km^2^⋅a)
−10000 ∼−6000 *(−7532.98* ± *265.24)*	0.53 ± 0.07	−85.35 ± 0.15	−5.53 ± 0.01	−5.08 ± 0.01
−5999 ∼−3500 *(*−*4482.55* ± *145.90)*	0.35 ± 0.04	−53.72 ± 0.09	−3.47 ± 0.01	−3.04 ± 0.001
−3500 ∼−500 *(*−*1645.46* ± *129.00)*	0.18 ± 0.03	−24.76 ± 0.06	−1.53 ± 0.001	−1.18 ± 0.001
−500 ∼ 200 *(*−*78.17* ± *20.93)*	0.11 ± 0.02	−1.75 ± 0.01	−0.09 ± 0.001	−0.06 ± 0.001
200 ∼ 2400 *(489.23* ± *35.96)*	0.16 ± 0.02	8.83 ± 0.02	0.51 ± 0.01	0.35 ± 0.001

*ΔErosion rate shows the erosion rate changes between the 1990s and 2010s expressed as a range (same as Figure 4A). The values are presented as the mean standard error. The values in italics are the changes in the average erosion rate. “−” indicates erosion rates are decreased in the 2010s compared with the 1990s.*

**FIGURE 6:**
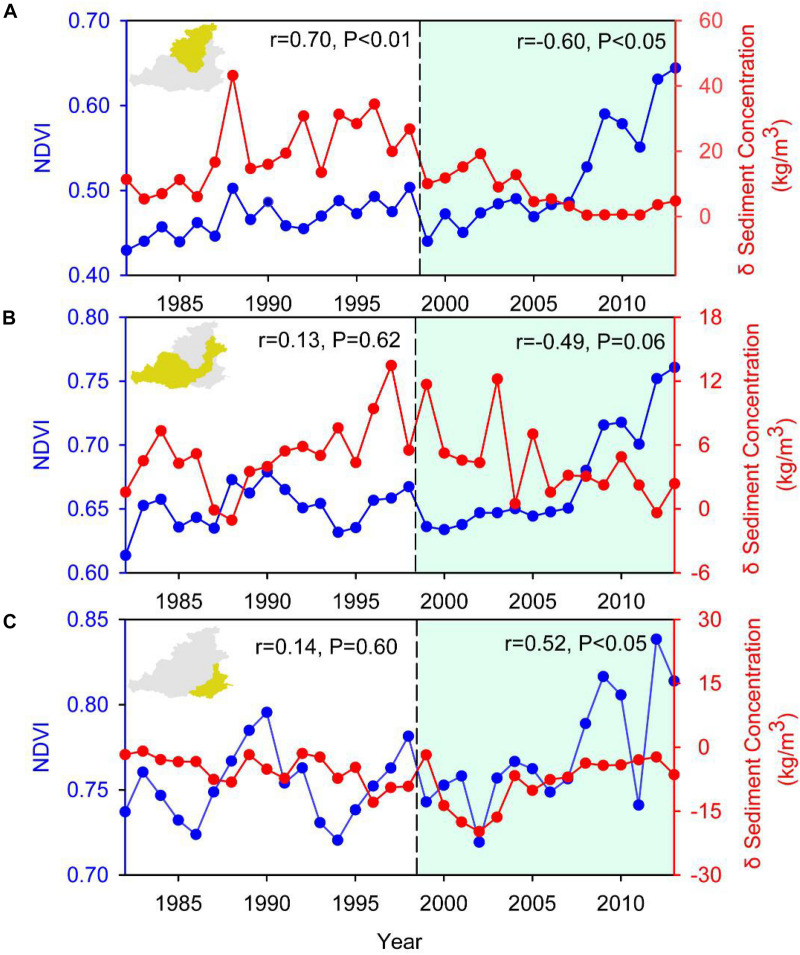
Relationships between the NDVI and sediment concentration at corresponding hydrologic stations. **(A)** The sediment concentration is the difference between Toudaoguai and Longmen. **(B)** The sediment concentration is the difference between Longmen and Tongguan. **(C)** The sediment concentration in the bottom panel is the difference between Tongguan and Huayuankou.

The differences of sediment concentration between two hydrologic stations reflected the soil erosion export in the sub-catchment. The three sub-catchments showed different patterns with respect to the relationship between vegetation changes and soil erosion export (Figure 6). In catchment I, before the implementation of the GFGP, the variation of vegetation changes was coincident with the soil erosion export because the vegetation cover was too low to prevent soil erosion export. However, after the implementation of the GFGP, the vegetation trend was opposite to the changes in sediment concentration, indicating that the planting of trees and grasses effectively prevented soil erosion export. In catchment II, vegetation changes had a poor relationship with the difference in sediment concentration before the GFGP, whereas there was a negative relationship between vegetation changes and differences in sediment concentrations after the GFGP (*R* = −0.49, *P* = 0.06). The patterns of vegetation and soil are complex because this region is a combined region of the GFGP (northern part) and human activities (southern part). In catchment III, a place with no GFGP activity and an increase in soil erosion export, vegetation changes and differences in sediment concentrations had no relationship before the GFGP. However, there was a positive relationship between these two indicators after the GFGP because the vegetation cover was too low to prevent soil erosion export.

Specific small watershed showed similar characteristics (Supplementary Figure S4). The Wuding River watershed is a classic region in which the GFGP was implemented in catchment I (Supplementary Figure S4A). Wuqi County in this region is one of most successful counties in implementing the GFGP. The relationship between vegetation changes and sediment concentrations in the Baijiachuan hydrologic station had a positive relationship before the GFGP, showing that vegetation cover was too low to prevent soil erosion export in this watershed. However, after the GFGP, the two variables showed an obvious negative relationship, indicating that planted vegetation played a strong role in preventing soil erosion export. In the watershed of the Fen River, a region of combined human activity and the GFGP, a weak relationship was observed between vegetation changes and sediment concentrations at Hejin station before the GFGP (Supplementary Figure S4B). After the GFGP, increasing vegetation greenness prevented soil erosion export, but this effect was not significant (Supplementary Figure S4B, *R* = 0.24, *P* = 0.38). Different from the catchment of the Fen River, the watersheds of the Wei and Jing rivers showed a positive relationship between the two variables after the GFGP (Supplementary Figure S4C) because of intense human activities.

## Discussion

### Vegetation Change and Its Drivers

The changes of vegetation cover are consistent with land transfer direction from cropland to forestland and grassland, which confirmed the GFGP is the main driver of vegetation changes. Before the GFGP, only 2.0% of the study area (6,725 km^2^) changed. The main land use transfer directions were from barren land to grassland and from grassland to cropland, which accounted for 18.6 and 14.8% of the changed area, respectively (Supplementary Figures S5–S7). The changes from barren land to grassland mainly occurred in the northwestern region, a region of desertification control in China (the blue polygon in Supplementary Figure S6A). During this period, a massive amount of grassland was converted to cropland to meet food supply demands. After the GFGP, 5.0% of the study area (17, 173 km^2^) changed. Conversions from cropland to forestland and grassland accounted for more than 56% of the changed areas, indicating that the main features of land use changes were caused by the GFGP. The great effects of the GFGP on increasing vegetation were also confirmed by afforestation data collected from the Chinese forestry annual reports (Supplementary Figure S8).

The contributions of climate and human activities to the increased vegetation after the GFGP were quantified. The area controlled by human activities accounted for 53% of the study area (Supplementary Figure S9). A case study on the northwestern Loess Plateau showed that afforestation would effectively increase regional precipitation and decrease the surface temperature (Wang et al., 2018). Our results also support the existence of interactions between climate (especially precipitation) and human activities (mainly the GFGP). These interactions occurred in the forest-grassland ecotone, accounting for 16% of the study area. The results showed that the areas regulated by the climate accounted for a small proportion of the study area (only 5%), which mainly occurred in the forestland (Supplementary Figure S9).

### Significance of the Vegetation Restoration Effects on Soil Erosion and Nutrient Loss

This study confirmed the contributions of improved vegetation cover to the reduce of soil erosion export and nutrient loss. According to our results, there is a significant relationship between soil erosion export and vegetation changes at the county level on the Loess Plateau. This work highlights that vegetation restoration has effectively reduced soil erosion and nutrient export. However, planting more trees or grass will consume more soil water. Some studies stated that vegetation expansion in water-limited areas, such as, the Loess Plateau, creates conflicting demands for water between ecosystems and humans (Farley et al., 2005; Feng et al., 2016). The next step of ecological construction in water-limited areas is not planting more trees but rather, improving forest quality through forest management and protecting natural ecosystems (Cao, 2008; Zhu et al., 2017).

This study divided the whole area into three sub-catchments, which showed spatial heterogeneities at a large scale. The three sub-catchments represent different extents of soil erosion export that provide a possible way to build relationships among vegetation restoration, soil erosion export and nutrient loss. The study periods were divided into before and after the implementation of the GFGP, which showed that great progress has been achieved on the Loess Plateau. Soil erosion and nutrient export in different regions and periods will also provide good validation data for regional models. This study has demonstrated that vegetation restoration has significant impacts on soil erosion export. Vegetation restoration has increased the soil C dynamics and improved the soil structure (Deng and Shangguan, 2017; Zhao et al., 2017a). The change in soil erosion was also related to precipitation, soil properties, topography, land use change, and management; however, these factors are relatively stable or can be reflected in the vegetation- soil erosion relationship.

The importance of vegetation cover and vegetation compositions in controlling soil erosion is widely accepted because it induces a previously unaccounted for terrestrial sink or source for atmospheric carbon dioxide (Berhe et al., 2018). On the one hand, soil erosion leads to a loss of soil organic matter through the direct removal of soil mass and affects the mechanisms of soil organic matter stabilization (Chappell et al., 2016). On the other hand, the process of soil erosion shapes the ground surface, changes the distribution of nutrients and landscapes and delivers sediments to rivers and oceans (Prosser et al., 2001; Bouwman et al., 2013). Moreover, the stoichiometric relationships of C, N and P are relatively stable; however, some studies found that erosion affects these relationships, which will greatly affect ecological processes (Quinton et al., 2010; Yuan and Chen, 2015).

### Mechanism of Vegetation Effects on Soil and Nutrient Export

The importance of plants in controlling soil erosion export is widely recognized. Generally, at the individual level, the role of plants in reducing soil erosion can be summarized into three aspects. First, before soil erosion, plants reduce the energy of raindrops with their canopy, therefore breaking the impact of a raindrop before it hits the soil, further reducing the soil’s susceptibility to erosion (Zuazo and Pleguezuelo, 2009; Vaezi et al., 2017). Second, during the process of soil erosion, soil is prevented from being swept or blown away by the plant roots that hold the soil in place and redesign the rhizosphere to alter the three−dimensional physical architecture and water dynamics as a physical barrier (Bashir et al., 2018; Rabbi et al., 2018), moreover, high root density can protect soil detachment and increase infiltration, thus reducing soil loss (Gyssels et al., 2005). Third, during the process of soil deposition and redistribution, the plants slow down water as it flows over the land, allowing much of the runoff to soak into the ground (Graham, 2017; Gachene et al., 2019).

In the community level, plant type, plant diversity, the vegetation structure and the distribution pattern also affect the processes of runoff, soil erosion and nutrient export. Liu et al. (2020) found the main vegetation types used in ecological restorations have different behaviors in reducing runoff and sediment yield, for example, grassland showed a higher performance in maintaining runoff yield and reducing sediment delivery compared to forestland and scrubland. Plant diversity can affect soil erosion and runoff by changing the pattern of vertical vegetation (Martin et al., 2010; Gómez et al., 2018), enhancing the light use efficiency of aboveground vegetation cover (Onoda et al., 2014) and increasing the diversity of the litter layer and root density (Zheng et al., 2017; Liu X. et al., 2018). The effects of the vegetation structure on rainfall include the aboveground layer, surface litter layer and belowground root layer. The aboveground vegetation layer redistributes rainfall into canopy interception, stemflow and throughfall, thereby weakening rainfall and controlling soil erosion (Muzylo et al., 2009; Allen et al., 2014). Moreover, the litter layer can intercept rainfall, increase infiltration, decelerate surface runoff, and therefore reduce soil loss (Pannkuk and Robichaud, 2003; Liu J. et al., 2018).

In the landscape level, the spatial distribution of vegetation can greatly affect runoff and sediment yields (Bautista et al., 2007; Wei et al., 2015). A change in vegetation will change the landscape connectivity and fragmentation level and will thus affect the surface runoff and sediment delivery (Fryirs, 2013). As vegetation becoming sparse, runoff and sediment yields increase (Imeson and Prinsen, 2004; Bautista et al., 2007). The relationship between vegetation cover and soil loss is also scale dependent, for example, at the patch or landscape level, we should pay more attention to the vegetation structure and plant diversity; at the catchment or regional scale, we should focus on the changes in vegetation cover.

### Challenges and Future Directions

Although the national surveys of soil erosion during the periods from 1995 to 1996 and 2010 to 2012 (Yue et al., 2016) represent the most credible data on the Loess Plateau, uncertainties remain. The first national survey (from 1995 to 1996) combined TM images and field survey data to provide spatial distribution information on primary geographical and environmental factors. The second national survey is calculated by Chinese Soil Loess Equation (CSLE, Liu et al., 2001) modeled with topographical, land use, and remote-sensing information as well as field survey data as input variables. Considering the uncertainties of variables of CSLE, the national survey data only provided soil erosion grades (slight, light, moderate, intense, extremely intense and severe erosion). We used the median value instead of the actual erosion rate, which brought much of the uncertainty. When we calculated the nutrient loss, we assumed that erosion did not influence the soil nutrient content that is problematic on the decade timescale (Yue et al., 2016).

There were some uncertainties when we simply calculated the process of soil erosion export as the differences between soil erosion and soil deposition, not considering the effects of time transgressive processes. First, soil erosion selectively removes the fine organic particles, leaving behind large particles and stones (Víctor and Carmen, 2008), and resulting the exposure of less erodible soil materials from deeper beneath the surface which might have been mildly cemented, for instance, by carbonates or other materials, during pedogenic processes (Zamanian et al., 2016; Vasu et al., 2018). Second, in the process of soil deposition, the soil nutrient facilitates the formation of soil aggregates and increased soil porosity, which changes soil structure and water infiltration (Liu et al., 2019). However, we assumed the soil nutrient content and soil erodibility remained unchanged before and after the GFGP and not considered effects of these processes. Moreover, the effects of extreme climate factors, including intense precipitation, on the evaluation of soil erosion were less considered in the study.

In future studies, we recommend three directions. The first is enhancing the observations of soil erosion processes at different scales, which will be helpful to improve the model accuracy of soil erosion at each process (Martinez et al., 2017). The second is paying more attention to the soil-atmosphere intersections, especially the greenhouse gas emission during the process of soil erosion (Bradford et al., 2016; Gao et al., 2018). The third is projecting the vegetation changes, soil erosion export and nutrient loss in the changing climate conditions (Pastor et al., 2019), which has a significant to biogeochemical cycles and will provide references to the policy-making for local government.

## Conclusion

Our work shows that vegetation restoration from 1999 to 2013 on the Loess Plateau has significantly reduced soil erosion and nutrient export. After the implementation of the GFGP, the vegetation greenness improved significantly, and soil erosion export was reduced to a great extent. A series of expressions related to the vegetation erosion—nutrient loss relationships were built for the Loess Plateau and are significant for ecological studies and modeling. Quantifying all the processes of soil erosion that provide more accurate descriptions of nutrient cycles requires more field experimental data in the future.

## Data Availability Statement

Publicly available datasets were analyzed in this study. The GIMMS NDVI-3g data can be found here: https://ecocast.arc.nasa.gov/data/pub/gimms/3g.v1/. The soil erosion data can be found in online repositories. The name of the repository and accession number can be found in Yue et al. (2016). The soil erosion data can be found in Yue et al. (2016). The land use map can be download from http://www.resdc.cn/. The data of the 0–30 cm soil nutrient content cam be download from http://www.fao.org/landwater/databases-andsoftware/hwsd/en/. The data of afforestation area is recorded in Chinese Forestry Annual Statistical Reports (Beijing, China Forestry Publishing House). The climate data can be found here: http://data.cma.cn/. The runoff and sediment data are recorded in River Sediment Bulletin of the Yellow River from 1982 to 2012 (http://www.yrcc.gov.cn/nishagonggao/).

## Author Contributions

JZ, XF, and BF designed the framework of the manuscript. JZ carried out all the statistical analyses and wrote the first draft of the manuscript. LD, YY, ZZ, PZ, and CP provided additional advice on the analysis. All authors provided input to the final draft.

## Conflict of Interest

The authors declare that the research was conducted in the absence of any commercial or financial relationships that could be construed as a potential conflict of interest.

## Supplementary Material

Supplementary Figure 1 Description of the technique used in this study.

Supplementary Figure 2 Total nutrient loss in the study area. **(A)** Total SOM loss. **(B)** Total N loss. **(C)** Total P loss.

Supplementary Figure 3 Spatial distributions of total nutrient losses in the 1990s and 2010s. **(A,B)** Total SOM loss. **(C,D)** Total soil nitrogen loss. **(E,F)** Total phosphorus loss.

Supplementary Figure 4 Relationship between the NDVI and sediment concentration at corresponding tributary catchments. **(A)** The catchment of the Wuding River; the corresponding hydrological station is Baijiachuan. **(B)** The catchment of the Fen River; the corresponding hydrological station is Hejin. **(C)** The catchment of the Wei River; the corresponding hydrological station is Huaxian.

Supplementary Figure 5 Land use types in 1980 **(A)**, 2000 **(B)**, and 2015 **(C)**.

Supplementary Figure 6 Land use changes before and after the Grain-for-Green Projects (GFGP). **(A)** From 1980 to 2000 (before the GFGP). **(B)** From 2000 to 2015 (after the GFGP). Top five land use transfer types are shown in the top-left panels. The percentages stand for the proportions in the changed area. Barren land (B); grassland (G); cropland (Cr); wetland (W); construction land (Co). The colors of map legend are consistent with the bars in panels.

Supplementary Figure 7 shows the photographs of red points: **(A)** (Xiaojihan) and **(B)** (Xindiangou).

Supplementary Figure 8 Photographs of afforestation areas (taken by Xiaojiang Zhai, Shaanxi Academy of Forestry). **(A)** Xiaojihan forest farm, Yuyang District, Yulin City, Shaanxi Province; **(B)** Xindiangou, Suide County, Yulin City, Shaanxi Province.

Supplementary Figure 9 Contributions of climate (annual precipitation, annual temperature and solar radiation) and human activities (mainly the GFGP) to increased vegetation during 2000–2013.
