# Supplementary material for: Quantifying the Effects of Vegetation Restorations on the Soil Erosion Export and Nutrient Loss on the Loess Plateau
Source: Front Plant Sci. 2020 Nov 27;11:573126. doi: 10.3389/fpls.2020.573126 (PMC7728691; doi:10.3389/fpls.2020.573126)

# SUPPORTING INFORMATION

**Quantifying the effects of vegetation restorations on the soil erosion export and nutrient loss on the Loess Plateau**

Jun Zhao^1^, Xiaoming Feng^1*^, Lei Deng^2^, Yanzheng Yang^1^, Zhong Zhao^3^, Pengxiang Zhao^3^, Changhui Peng^3,4^, Bojie Fu^1^

^1^ State Key Laboratory of Urban and Regional Ecology, Research Center for Eco-environmental Sciences, Chinese Academy of Sciences, Beijing 100085, China

2 Institute of Soil and Water conservation, Northwest A&F University, Yangling, Shaanxi 712100, China

^3^ College of Forestry, Northwest A&F University, Yangling, Shaanxi 712100, China

^4^ Department of Biology Sciences, Institute of Environment Sciences, University of Quebec at Montreal, Montreal, Canada

Corresponding Author: Prof. Xiaoming Feng ([fengxm@rcees.ac.cn](mailto:fengxm@rcees.ac.cn))

To be submitted to “***Frontiers in Plant Science***”

**Figure S1** Description of the technique used in this study.

**
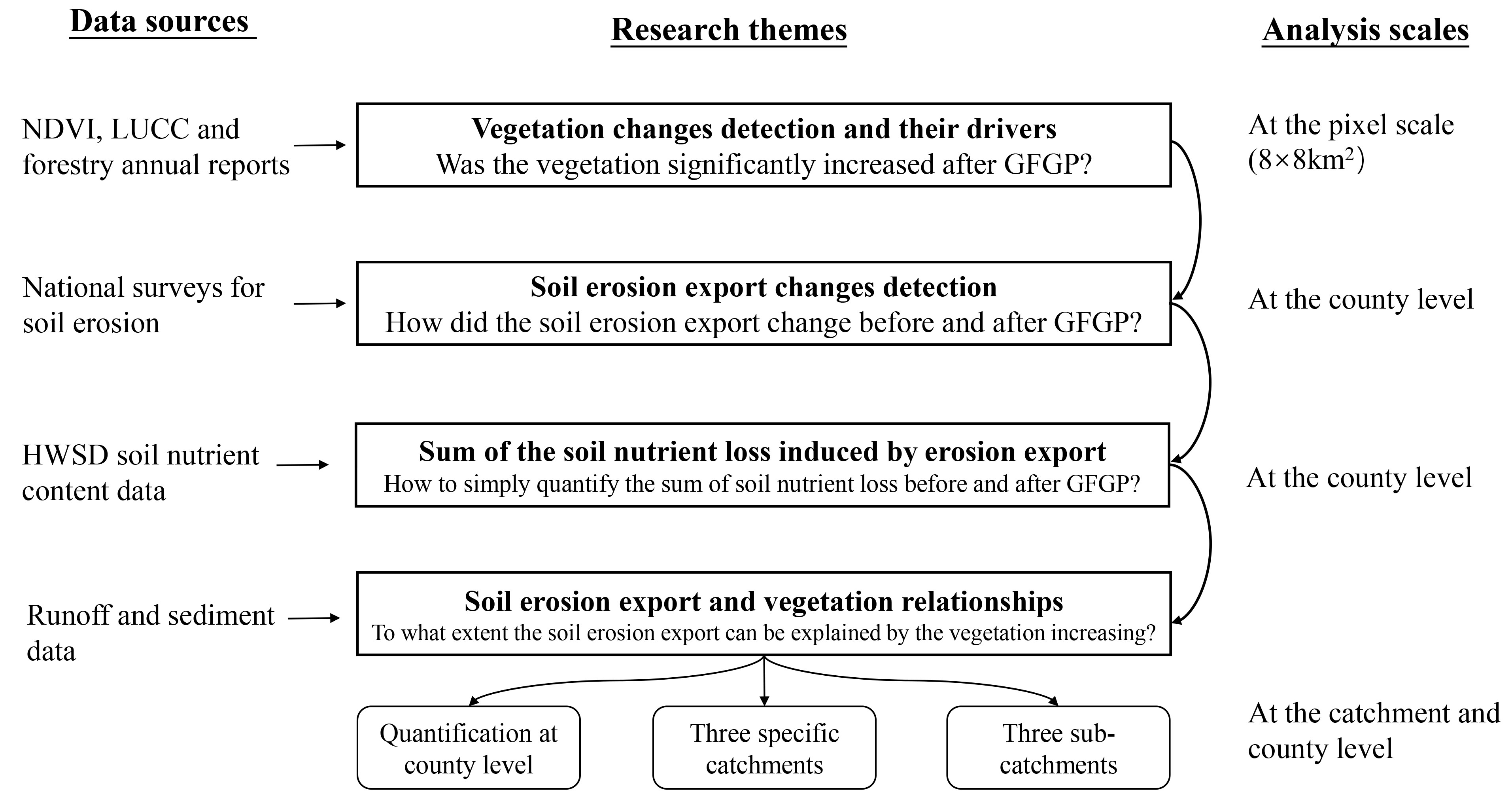
**

**Figure S2** Total nutrient loss in the study area. (a) Total SOM loss. (b) Total N loss. (c) Total P loss.


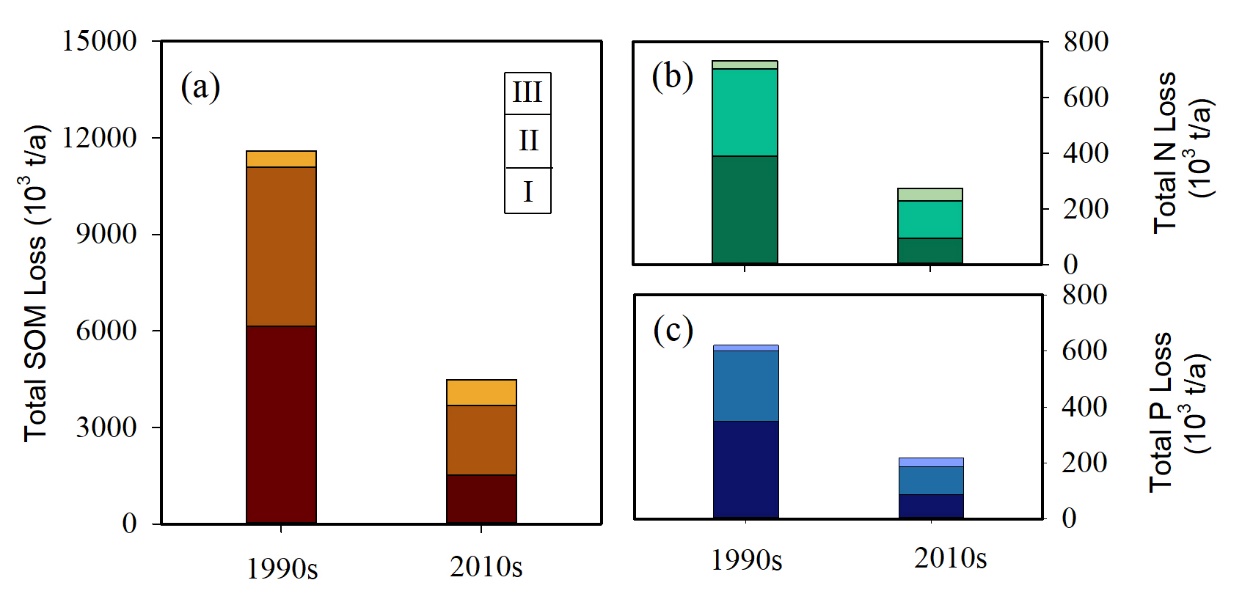


**Figure S3** Spatial distributions of total nutrient losses in the 1990s and 2010s. (a-b) Total SOM loss. (c-d) Total soil nitrogen loss. (e-f) Total phosphorus loss.


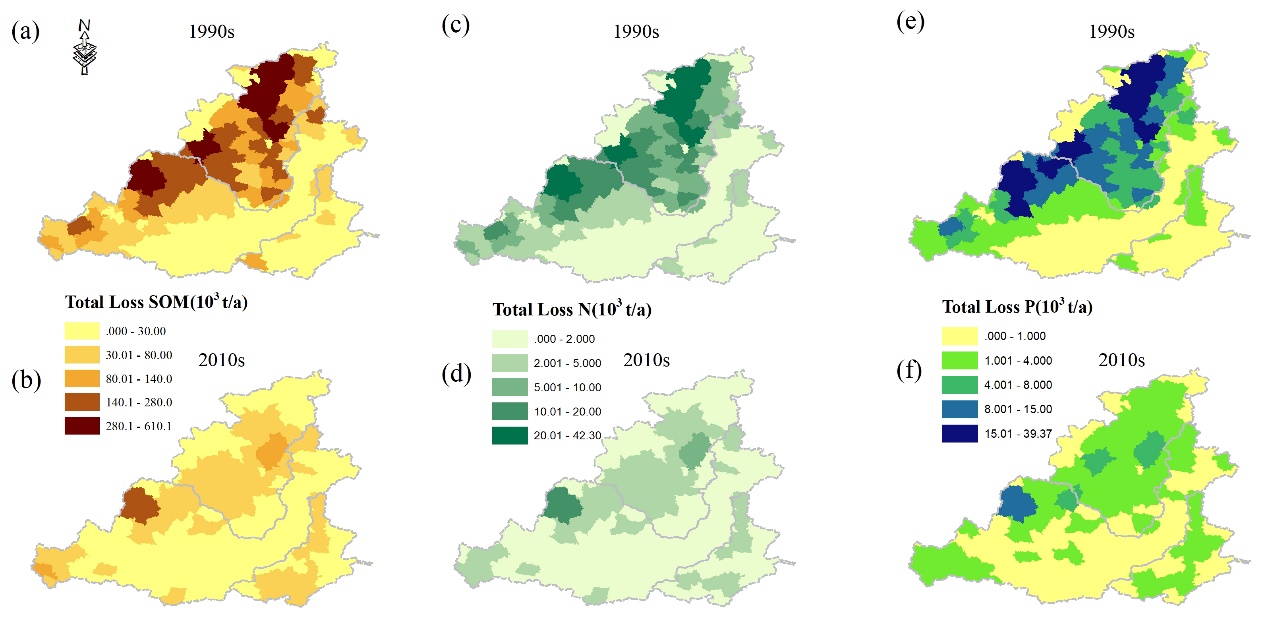


**Figure S4** Relationship between the NDVI and sediment concentration at corresponding tributary catchments. (a) The catchment of the Wuding River; the corresponding hydrological station is Baijiachuan. (b) The catchment of the Fen River; the corresponding hydrological station is Hejin. (c) The catchment of the Wei River; the corresponding hydrological station is Huaxian.


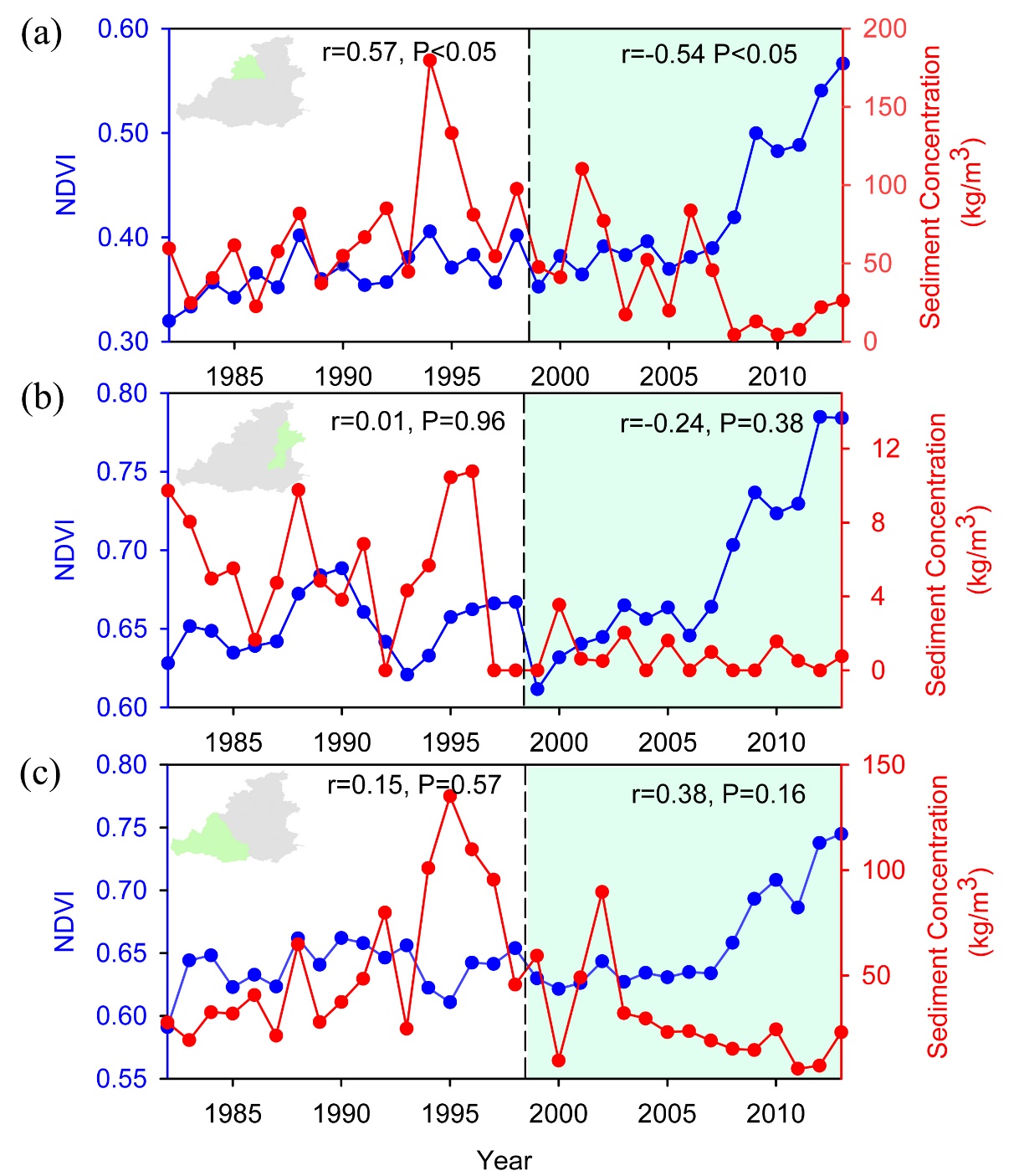


**Figure S5** Land use types in 1980(a), 2000(b) and 2015(c).

**
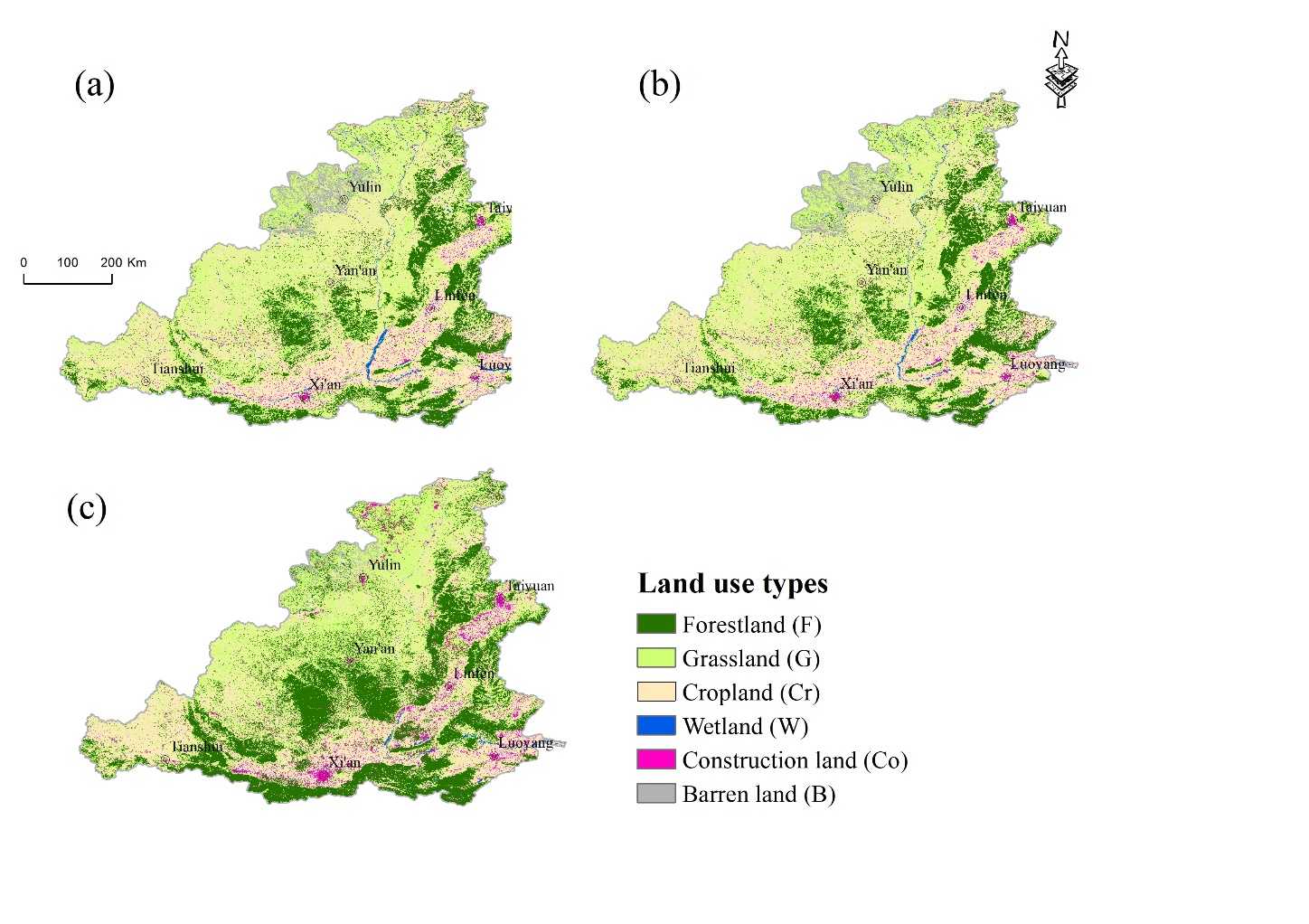
**

**Figure S6** Land use changes before and after the Grain-for-Green Projects (GFGP). (a) From 1980 to 2000 (before the GFGP). (b) From 2000 to 2015 (after the GFGP). Top five land use transfer types are shown in the top-left panels. The percentages stand for the proportions in the changed area. Barren land (B); grassland (G); cropland (Cr); wetland (W); construction land (Co). The colours of map legend are consistent with the bars in panels. Figure S7 shows the photographs of red points: A (Xiaojihan) and B (Xindiangou).


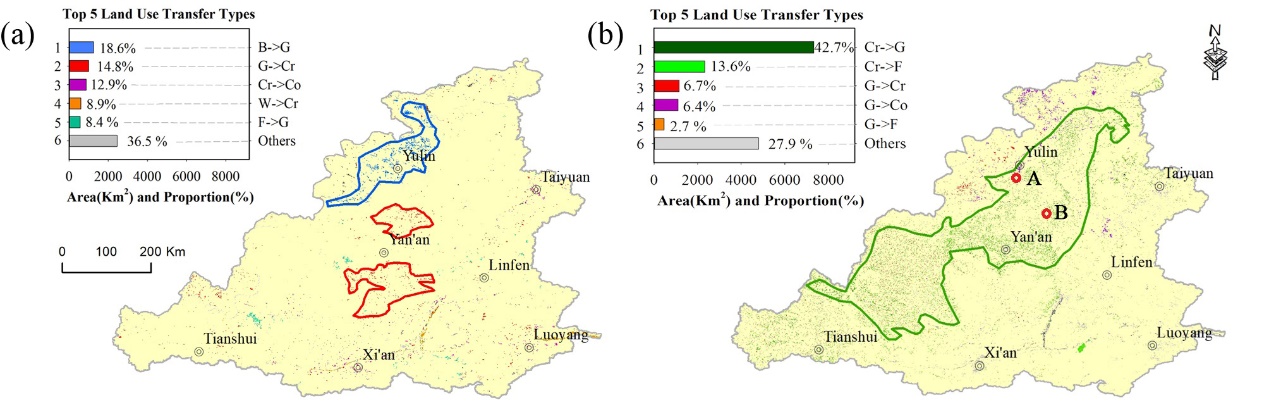


**Figure S7** Photographs of afforestation areas (taken by Xiaojiang Zhai, Shaanxi Academy of Forestry). (A) Xiaojihan forest farm, Yuyang District, Yulin City, Shaanxi Province; (B) Xindiangou, Suide County, Yulin City, Shaanxi Province

**
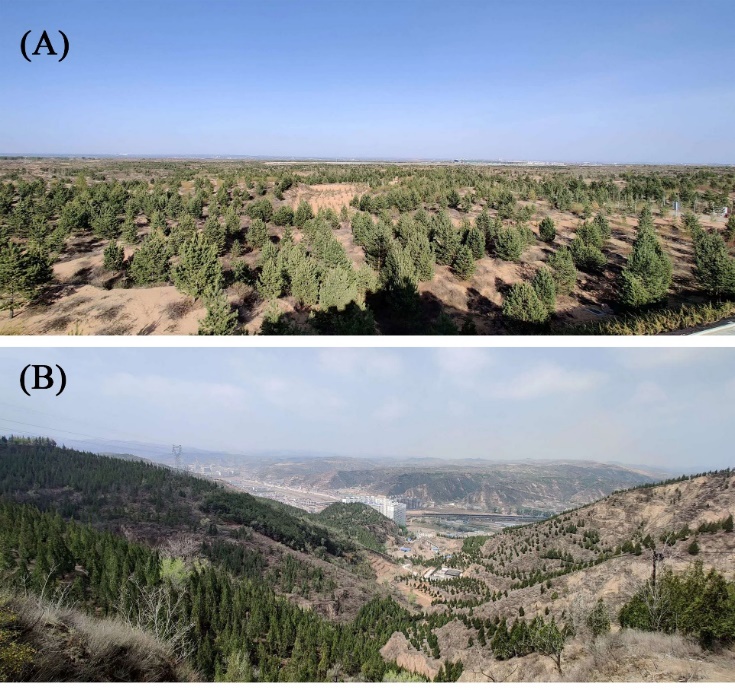
**

**Figure S8** Annual afforestation areas for each county on the Loess Plateau from 2002 to 2013 (data from Chinese forestry annual reports).


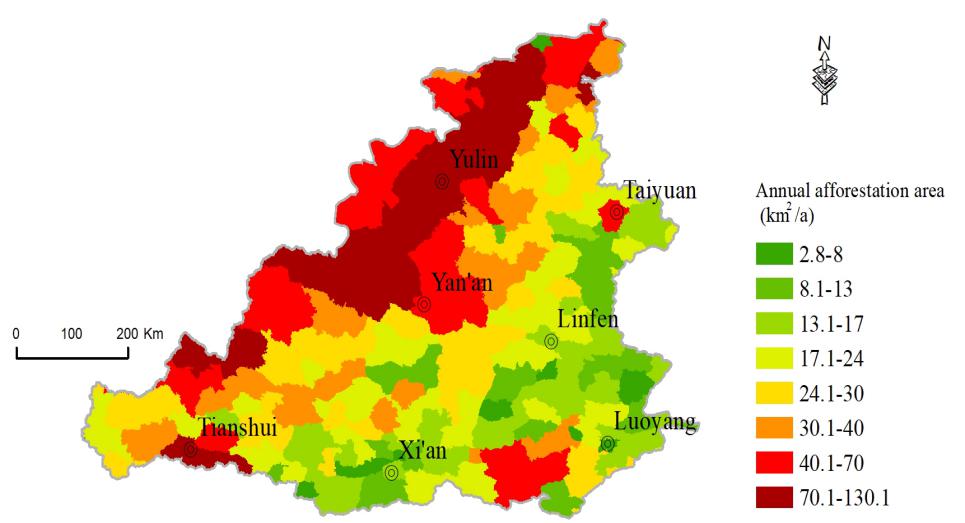


**Figure S9** Contributions of climate (annual precipitation, annual temperature and solar radiation) and human activities (mainly the GFGP) to increased vegetation during 2000-2013.


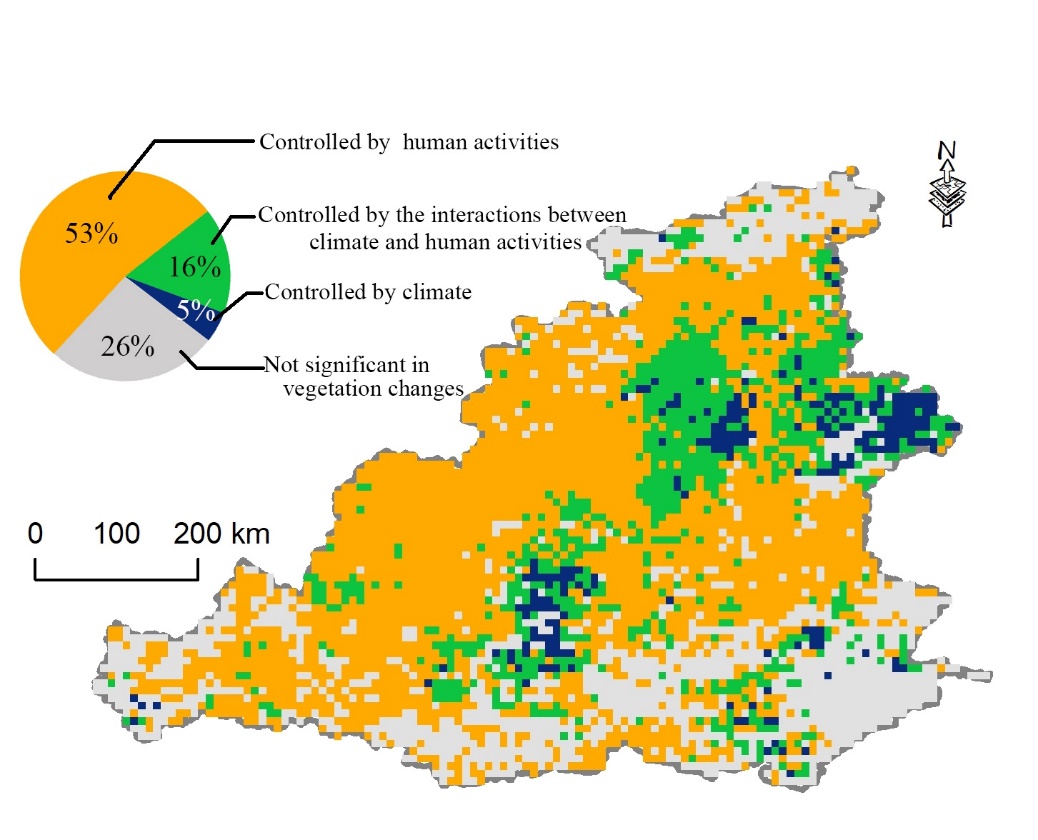

Supplement: Supplementary Figure 1 — Description of the technique used in this study. [file Data_Sheet_1.docx]
